# Rising atmospheric CO_2_ outweighs elevated vapor pressure deficit in explaining increased intrinsic water use efficiency in Chinese pine

**DOI:** 10.3389/fpls.2026.1721057

**Published:** 2026-04-01

**Authors:** Yuanqiao Li, Yunni Wang, Wenfang Xu, Jinkai Tan

**Affiliations:** 1 College of Forestry, Fujian Agriculture and Forestry University, Fuzhou, China; 2 Inner Mongolia Academy of Forestry Sciences, Hohhot, China; 3 South China Botanical Garden, Chinese Academy of Sciences, Guangzhou, China; 4 School of Artificial Intelligence, Shenzhen Technology University, Shenzhen, China

**Keywords:** intrinsic water use efficiency, atmospheric CO_2_, vapor pressure deficit, soil moisture, Chinese pine (*Pinus tabulaeformis*)

## Abstract

**Introduction:**

Tree intrinsic water use efficiency (iWUE) is an important metric for carbon and water balance in forest ecosystems. Tree iWUE has widely increased due to rising atmospheric CO_2_ and intensified drought. However, the dominant effects of rising atmospheric CO_2_2, vapor pressure deficit (VPD), and soil moisture on iWUE are not fully understood.

**Methods:**

We developed tree-ring width, stable carbon and oxygen isotopic chronologies derived from Chinese pine (*Pinus tabulaeformis*) stands across northern China to quantify the ecophysiological responses of iWUE to atmospheric CO_2_ and drought.

**Results:**

iWUE was significantly correlated with atmospheric CO_2_ and VPD at both dry and wet sites. Furthermore, increased assimilation rate led to increased iWUE at both dry and wet sites. Notably, the beneficial effect of atmospheric CO_2_ on iWUE outweighed that of VPD in Chinese pine.

**Discussion:**

Our findings highlight the dominant role of rising atmospheric CO_2_ in enhancing assimilation rate and increasing iWUE of Chinese pine and will aid in defining the performance of this tree species under climate change in northern China.

## 1 Introduction

How trees respond to climate change has a profound impact on the carbon and water balances in forest ecosystems (Mathias and Thomas, 2021), as trees control stomata to regulate the trade-off between carbon gain and water loss. Tree intrinsic water use efficiency (iWUE), defined as the ratio of assimilation rate to stomatal conductance, reflects the balance in carbon gain and water loss in forest ecosystems (Li et al., 2023; Martinez-Sancho et al., 2018; Panthi et al., 2020; Rodriguez-Caton et al., 2021). Increased tree iWUE has been reported in response to climate change and rising atmospheric CO_2_ (Adams et al., 2020; Keenan et al., 2013; Mathias and Thomas, 2021). However, significant uncertainties remain regarding the environmental drivers of iWUE, and the relative contribution of increased carbon assimilation versus decreased stomatal conductance to increased iWUE may vary across diverse climate regions.

There are several mechanisms that can potentially explain the increase in iWUE with increasing CO_2_. The combined impact of rising atmospheric CO_2_, warming, and intensified droughts has led to the increase of tree iWUE (Kannenberg et al., 2021; Keenan et al., 2013; Mathias and Thomas, 2021; Rahman et al., 2020). On the one hand, rising atmospheric CO_2_ could alter stomatal conductance by modifying stomatal density and shape. On the other hand, it could facilitate CO_2_ diffusion within the mesophyll and thereby promote photosynthesis (Gardner et al., 2023; Lauriks et al., 2021; Walker et al., 2021). These two processes could reduce transpiration while maintaining or enhancing vegetation productivity, increasing iWUE (Farquhar et al., 1982). Warming-induced drought may dampen the positive acclimation responses of leaf photosynthesis to rising atmospheric CO_2_ and increase iWUE if plants close their stomata to relieve water stress (Birami et al., 2020; Hao et al., 2023). However, whether rising atmospheric CO_2_ compensates for warming-induced decreasing water availability to sustain iWUE requires further quantification (Jia et al., 2022; Yang et al., 2021).

High vapor pressure deficit (VPD) and low soil moisture have been recognized as two main drought-related constraints on terrestrial water use and carbon uptake (Grossiord et al., 2020; Novick et al., 2024). Elevated VPD directly limits stomatal conductance, whereas soil moisture serves as the primary water source for tree uptake (Grossiord et al., 2020; Novick et al., 2024). Under low water availability (high VPD or low soil moisture), trees typically reduce stomatal conductance to minimize water loss, thereby increasing iWUE. However, the relative importance of VPD versus soil moisture in deriving iWUE variability remains inadequately quantified, a critical knowledge gap for predicting long-term physiological responses to intensifying drought conditions (Rahman et al., 2020). These regulatory effects likely vary across climatic zones and species (Dietrich et al., 2021; Jia et al., 2022; Thomas et al., 2018; Yi et al., 2019). Critically, VPD and soil moisture respond differentially to climate change: VPD exhibits high sensitivity to rising temperature and is projected to increase globally in the future, whereas precipitation and soil moisture changes are more uncertain, spatially heterogeneous, and relatively muted (Dai, 2013). Thus, disentangling their individual effects on iWUE is essential for understanding tree physiological adaptation under future climate change (Ficklin and Novick, 2017; Novick et al., 2016).

Tree rings have been widely employed to evaluate climatic and environmental impacts on forest ecosystems (Adams et al., 2020; Gessler et al., 2014; Li et al., 2023; Panthi et al., 2020). They not only provide annual records of radial growth but also preserve carbon (δ^13^C) and oxygen (δ^18^O) isotopes, offering insights into historical tree physiological responses to climatic and site conditions (Lin et al., 2019; Thomas et al., 2018; Vitali et al., 2021). iWUE can be derived from tree ring δ^13^C (McCarroll and Loader, 2004), whereas δ^18^O serve as proxies for stomatal conductance variation independent of assimilation rate (Mathias and Thomas, 2021; Pu and Lyu, 2023). The combinations of trends in δ^13^C and δ^18^O can be used to interpret the variations of stomatal conductance and assimilation rate, helping identify effects of stomatal conductance and assimilation rate on iWUE dynamics (Gessler et al., 2018; Scheidegger et al., 2000). Previous studies have demonstrated the important role of atmospheric CO_2_ in regulating iWUE (Keenan et al., 2013; Mathias and Thomas, 2021). However, few investigations have comprehensively assessed the impacts of VPD and soil moisture on the regulation of iWUE, and even fewer have examined the underlying physiological components—assimilation rate and stomatal conductance—driving these changes (Wang et al., 2024; Yi et al., 2019).

This study aims to quantify effects of atmospheric CO_2_, VPD, and soil moisture on iWUE in Chinese pine (*Pinus tabulaeformis*), a key afforestation species in drought-intensified northern China (Zhou et al., 2017; Zhu and Song, 2021). By examining photosynthetic assimilation rate and stomatal conductance across dry and wet sites, we test the following hypotheses: 1) rising atmospheric CO_2_ plays a dominant role in regulating the iWUE in both dry and wet sites; 2) VPD and soil moisture co-regulate the variation of iWUE in dry regions, whereas VPD predominantly controls the variation of iWUE in wet regions; 3) decreased stomatal conductance primarily drives increased iWUE in dry sites, whereas enhanced assimilation rate principally elevates iWUE in wet sites. The findings will advance physiological understanding of Chinese pine adaptivity to climate change.

## 2 Materials and methods

### 2.1 Study area

**Figure 1:**
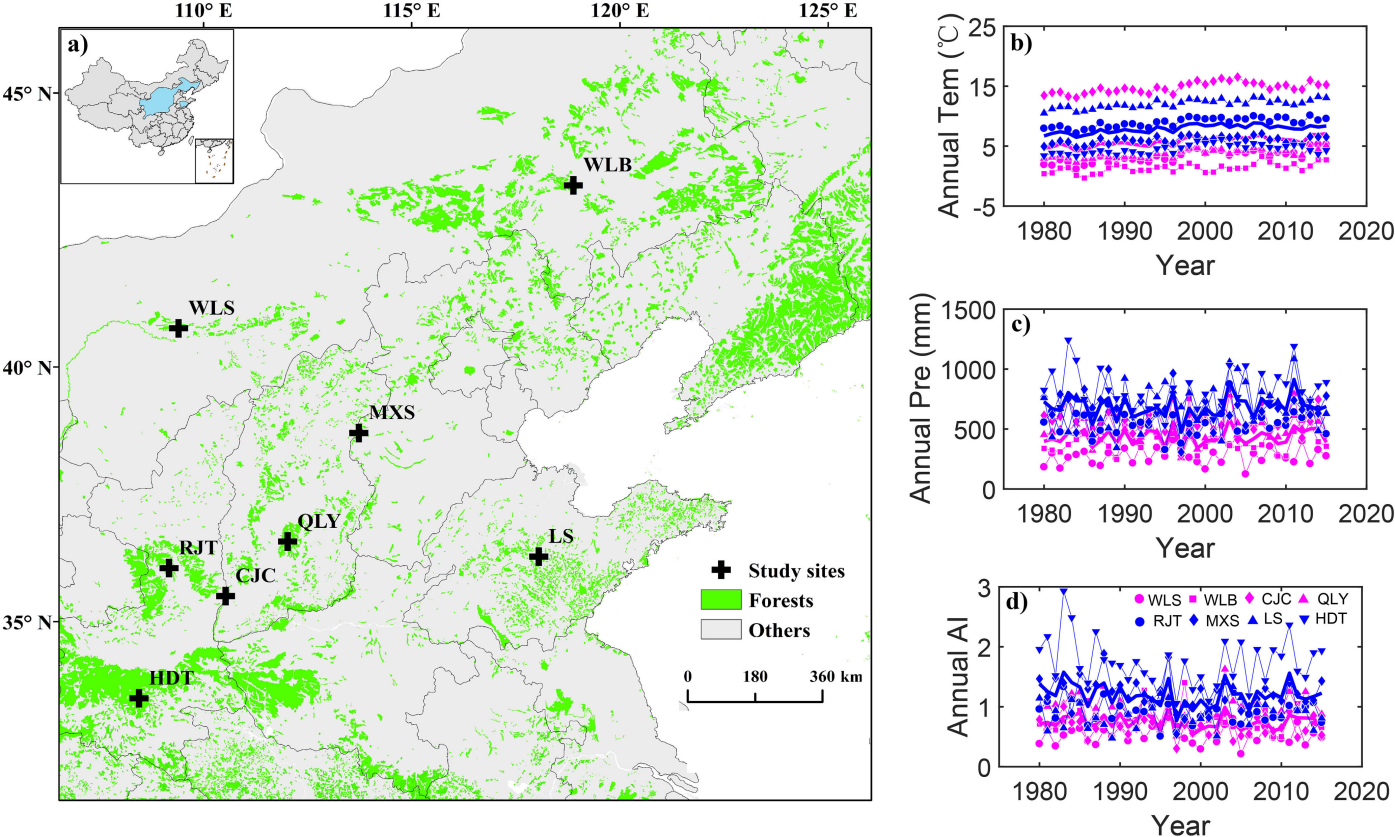
Geographical location **(a)** of study sites across northern China, and temporal variations of annual temperature [Tem, **(b)**], annual precipitation [Pre, **(c)**], and annual aridity index [AI, **(d)**] of Chinese pine in eight sites over northern China for the period of 1980–2015. The crosses in **(a)** represent the sampling sites, and the blue shade in the inset map is the distribution range of Chinese pine over northern China. Red colors in **(b–d)** represent dry sites, whereas blue colors represent wet sites. Different symbols represent different sites.

**Figure 2:**
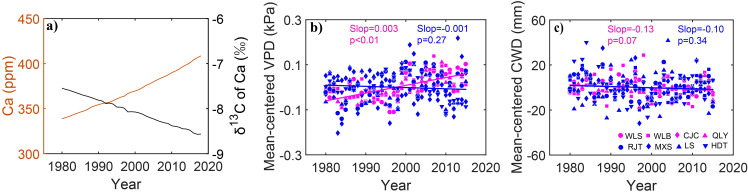
Temporal variations of the atmospheric CO_2_ and stable carbon isotopic values of air [Ca, orange line, δ^13^C of Ca, black line, **(a)**], the mean-centered vapor pressure deficit [VPD, **(b)**], and climatic water deficit [CWD, **(c)**] of Chinese pine in eight sites over northern China for the period of 1980–2015. Red colors represent dry sites, whereas blue colors represent wet sites. Different symbols represent different sites. The lines are the linear fits.

The study area covers the territory of Chinese pine distribution in northern China (**Figure 1**). This region experiences a typical East Asian monsoon climate, with >60% of annual precipitation concentrated between June and September. We established eight plots (25 m×25 m each) along a precipitation gradient spanning 208–814 mm. Mean annual temperature ranged from 1.38 °C to 12.12 °C, with monthly maximum temperature occurring in July–August and minimum temperature in December–January. Overall, based on the threshold of 529-mm annual precipitation for drought-limited growth of Chinese pine (Zhao et al., 2021), these plots were classified into dry sites (<529 mm) and wet sites (>529 mm). Specifically, the CJC site was classified as a dry site owing to its low aridity index. Detailed information regarding the fundamental physical and tree characteristics of each plot was recorded (**Figures 1**, **2**; **Table 1**).

**Table 1:** Chinese pine stand characteristics at the studied sites.

Group	Sites	Latitude (°)	Longitude (°)	MAT (°C)	MAP (mm)	MAI	Elevation (m)	Slope (°)	Height (m)	DBH (cm)	Density (trees/ha)
Dry sites	WLS	40.73	109.40	3.45	279	0.53	1930	26	7.13	18.50	169
	WLB	44.66	119.18	1.38	404	0.79	964	0	9.63	21.75	976
	CJC	35.52	110.53	8.83	563	0.67	1287	–	15.20	17.4	875
	QLY	36.61	112.02	3.82	487	0.87	1580	29	21.08	27.11	1936
Wet sites	RJT	36.08	109.17	8.95	561	0.91	1147	25	19.09	19.29	1776
	MXS	38.74	113.73	6.02	688	1.22	1347	18	7.70	15.71	1296
	LS	36.31	118.05	12.12	700	0.94	907	31.5	10.28	28.01	576
	HDT	33.43	108.45	4.61	814	1.73	1550	29	22.72	24.43	656

MAT, MAP, and MAI are the mean annual temperature, mean annual precipitation, and mean annual aridity index, respectively, during the period 1980–2015. DBH, diameter at breast height. “-” indicates the data are not available.

### 2.2 Tree ring sampling and process

In each plot, two perpendicular cores were extracted at breast height (1.3 m) from all trees >5 cm diameter at breast height for dendrochronological analysis. Cores were mounted, progressively polished with sandpaper (from coarse to fine grit), visually cross-dated, and measured to 0.01-mm precision using the LINTAB measuring system (Rinntech, Heidelberg, Germany). The quality of cross-dating and measurement was verified using the COFECHA program; the number of cores at each sites is shown in **Table 2**.

**Table 2:** Characteristics of the cross-dated tree-ring width and isotope series for the sample sites.

Group	Sites	Cores/trees	BAI time span	Isotope cores	Isotope data time span	Rbar
Dry sites	WLS	71/36	1955–2015	4	1972–2015	0.738 (δ^13^C)
	WLB	71/36	1977–2015	4	1980–2015	0.908 (δ^13^C)
	CJC	40/21	1975–2018	4	1975–2018	0.823 (δ^13^C)
	QLY	54/27	1959–2017	4	1966–2017	0.794 (δ^13^C), 0.725 (δ^18^O)
Wet sites	RJT	72/37	1979–2017	4	1980–2017	0.844 (δ^13^C)
	MXS	48/25	1965–2017	3	1970–2017	0.597 (δ^13^C)
	LS	46/24	1968–2018	3	1974–2017	0.857 (δ^13^C)
	HDT	37/20	1962–2017	4	1966–2017	0.649 (δ^13^C), 0.811 (δ^18^O)

BAI is basal area increment. Rbar means correlation with master series. δ^13^C means carbon isotopes series, and δ^18^O means oxygen isotope series.

Tree ring width measurements were converted into basal area increment (BAI) based on the distance between the innermost measured ring and the tree’s pith, using the package dplR. The raw ring-width series of each tree were converted into BAI (mm^2^ per year) using the following equation:


$$BAI=\text{π}(R_{t}^{2}-R_{t-1}^{2})$$

where R is the tree radial radius and t is the year of tree ring formation.

### 2.3 Climate data

Monthly air temperature, precipitation, specific humidity, and air pressure for the eight study sites were obtained from the China meteorological forcing dataset (https://data.tpdc.ac.cn/zh-hans/disallow/8028b944-daaa-4511-8769-965612652c49/). This dataset spans 1979–2018 with 0.1° spatial resolution and is widely recognized for its consistent quality and continuous temporal coverage (He et al., 2020).

Monthly VPD was calculated from air temperature and specific humidity using the Magnus equation (Murray, 1967; Zhou et al., 2020).


$$VPD=e_{s}-e$$

$$e_{s}=6.11\text{exp}(\frac{17.27\times Tem}{Tem+237.3})$$

$$e=\frac{qP}{\left(0.62+0.38q\right)}$$

where e_s_ is the saturation vapor pressure (hPa), e is the actual vapor pressure (hPa), Tem is temperature (°C), q is the specific humidity (kg/kg), and P is the air pressure.

As a key indicator of drought impacts on plant water stress, climate water deficit (CWD) is widely used to assess aridity and to predict vegetation growth. Here, monthly CWD was calculated from precipitation (Pre) and potential evapotranspiration (PET) to assess climatic water balance and soil moisture variations, as direct soil moisture measurements were not available. In addition, annual aridity index (AI) is the annual precipitation over the potential evapotranspiration, and higher aridity index represents wetter condition.


CWD=Pre−PET

AI=Pre/PET

where PET was calculated using the Thornthwaite equation (Thornthwaite, 1948) based on monthly temperature and site latitude, implemented through the SPEI package of R language (Beguería et al., 2014).

### 2.4 Stable δ^13^C and δ^18^O analysis

Following cross-dating and ring-width measurements, three to four cores with clear, regular ring boundaries and no missing rings from different trees were selected per site for δ^13^C analysis (**Table 2**). At two sites (QLY and HDT), the cores were additionally analyzed for δ^18^O. α-Cellulose was extracted directed from the annual tree ring using an improved plate method (Li et al., 2011).

δ^13^C and δ^18^O were measured using an isotope ratio mass spectrometer (Finnigan MAT Delta V advantage, Thermo Finnigan, San Jose, CA, USA). Isotope ratios are expressed as per mille deviations relative to Vienna Pee Dee Belemnite (δ^13^C) and Vienna Standard Mean Ocean Water (δ^18^O) standards. The within-run isotopic precision using quality control standards was 0.2‰ for δ^13^C and 0.3‰ for δ^18^O.

The carbon isotopic discrimination (Δ, ‰) was calculated as:


$$\Delta =(\delta^{13}Ca-\delta^{13}C_{tree})/(1000+\delta^{13}C_{tree})\times 1000$$

where δ^13^Ca and δ^13^C_tree_ are the isotope ratios of carbon in atmospheric CO_2_ and tree ring cellulose, respectively.

Δ is related to the intercellular CO_2_ (Ci) and atmospheric CO_2_ (Ca) by the following equation:


Δ=a+(b−a)(Ci/Ca)

where a and b are the fractionation factors during intercellular diffusion (4.4‰) and carboxylation (27‰).

The ratio of Ci to Ca was calculated following Farquhar et al. (1982):


$$Ci/Ca=[(\delta^{13}C_{tree}-\delta^{13}Ca+a)/(a-b)]$$

The iWUE, a proxy for physiological coordination ratio of assimilation rate (A) and stomatal conductance (g) (Farquhar et al., 1982; Gessler et al., 2014), was calculated as:


$$iWUE=\frac{\text{A}}{\text{g}}=(\text{Ca}-Ci)/1.6=Ca(1-\frac{Ci}{Ca})/1.6$$

Historic δ^13^Ca and Ca values were obtained from McCarroll and Loader (2004) up to year 2004 (http://www.esrl.noaa.gov/gmd/) and after that (2004–2017) were derived from the Mauna Loa Observatory of America (http://www.esrl.noaa.gov/gmd/obop/mlo/).

Leaf water δ^18^O enrichment above the source water (Δ^18^O_lw_) exhibits an inverse relationship with stomatal conductance (Mathias and Thomas, 2021). Leaf water δ^18^O enrichment above the source water was calculated from tree ring δ^18^O enrichment above the source water (Δ^18^O_tr_) and the oxygen isotopic composition of precipitation during ring formation (δ^18^O_p_) using the following equations:


$$\delta^{18}O_{p}=0.52\times MAT-0.006\times MAT^{2}+2.42\times MAP-1.43\times MAP^{2}-0.046\times \sqrt{E}-13$$

$$\Delta^{18}O_{tr}=\frac{\delta^{18}O_{tr}-\delta^{18}O_{P}}{1+\delta^{18}O_{P}/1000}$$

$$\epsilon_{wc}=0.0084\times MAT^{2}-0.51\times MAT+33.172$$

$$\Delta^{18}O_{lw}=\frac{\Delta^{18}O_{tr}-\epsilon_{wc}}{1-p_{x}p_{ex}}$$

where MAT is the mean annual temperature (°C), MAP is the total annual precipitation (m), E is the elevation (m) of the study location above sea level, ϵ_wc_ is the temperature-dependent fraction associated with carbonyl oxygen atoms exchanging with water during wood synthesis, p_x_p_ex_ approximately equal to 0.4 (Mathias and Thomas, 2021).

### 2.5 Statistical methods

Linear regression was adopted to analyze the temporal trends of the mean-centered VPD and soil moisture, iWUE, and leaf water δ^18^O enrichment above the source water (calculated by original values subtracting the mean values) during 1980–2015. Linear mixed-effects models were applied to identify drivers of iWUE and BAI variation with fixed-effect variables: atmospheric CO_2_, mean VPD, and climatic water deficit in biological year (from the previous October to the current September) during 1980–2015 and random effect variables: year and site. Only random intercepts were included in the model. Prior to modeling, iWUE, BAI, and fixed-effect variables were detrended by removing linear trends and standardized separately for dry and wet sites. Variance inflation factors of three drivers were calculated to assess collinearity, and collinearity was negligible, since variance inflation factors <3. The residuals followed a normal distribution, and the assumption of homogeneity of variance was satisfied across all four models. Random forest analysis was employed to assess the relative importance of environmental factors. In the random forest analysis, the training data account for 70% of available data; the remaining 30% of data was used for testing at dry sites and wet sites. A grid search was used to find the two optimal parameters, which is the number of trees and the number of input features through the randomForest package of R language, and the two parameters were set to 1,000 and 3, respectively. The larger values of increase in mean squared error mean the greater importance of the variables. Finally, a dual-isotope conceptual model was performed to illustrate variation in assimilation rate and stomatal conductance (Scheidegger et al., 2000).

## 3 Result

### 3.1 Temporal trends in environmental factors

A significant increasing trend in atmospheric CO_2_ and a decreasing trend in stable carbon isotopic values of air were observed during 1980–2015. VPD showed an obvious rise at all sites during 1980–2015, with a higher increased rate at dry sites (0.004 kPa/a) than that at wet sites (0.003 kPa/a). In addition, climatic water deficit displayed a decrease during 1980–2015, which decreased significantly at wet sites compared with dry sites (**Figure 2**).

### 3.2 Temporal trends in iWUE and environmental responses

**Figure 3:**
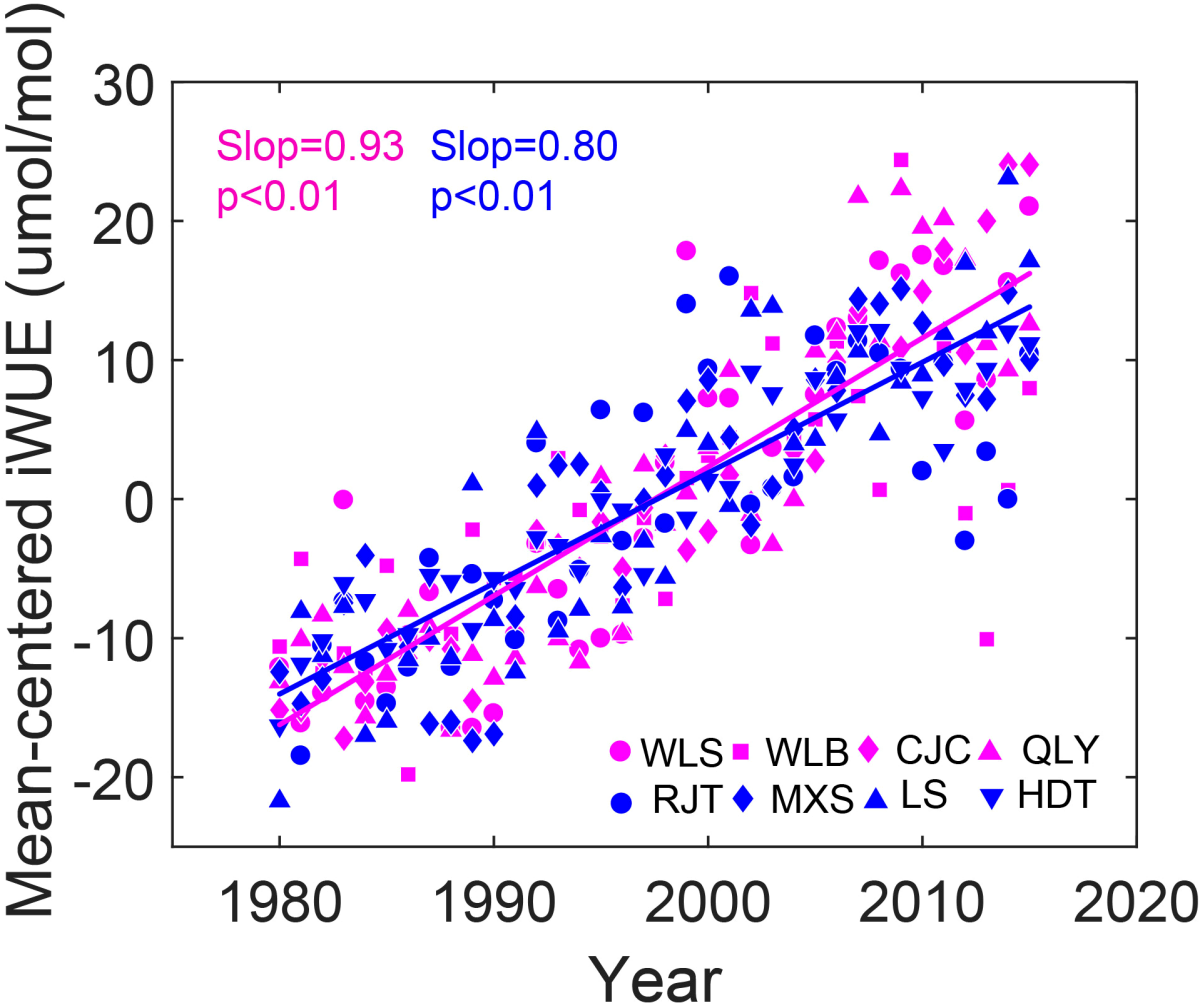
Temporal variations of mean-centered intrinsic water use efficiency (iWUE) of Chinese pine in eight sites over northern China for the period of 1980–2015. Red colors represent dry sites, whereas blue colors represent wet sites. Different symbols represent different sites. The lines are the linear fits.

**Figure 4:**
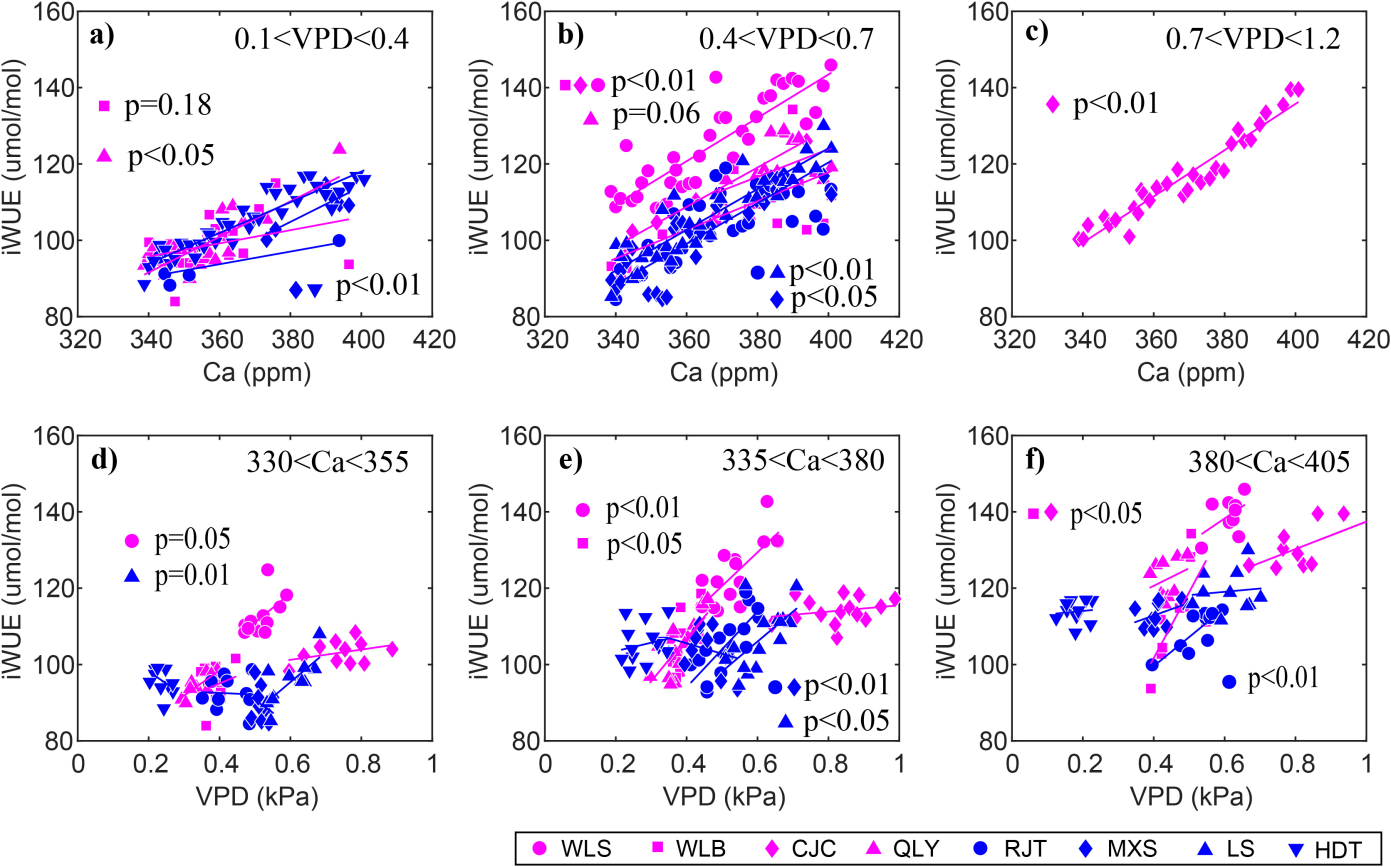
Relationships between environmental drivers [**(a–c)** atmospheric CO_2_ (Ca), **(d–f)** vapor pressure deficit (VPD)] and intrinsic water use efficiency (iWUE) in different groups. Different symbols represent different sites. The lines are the linear fits.

**Figure 5:**
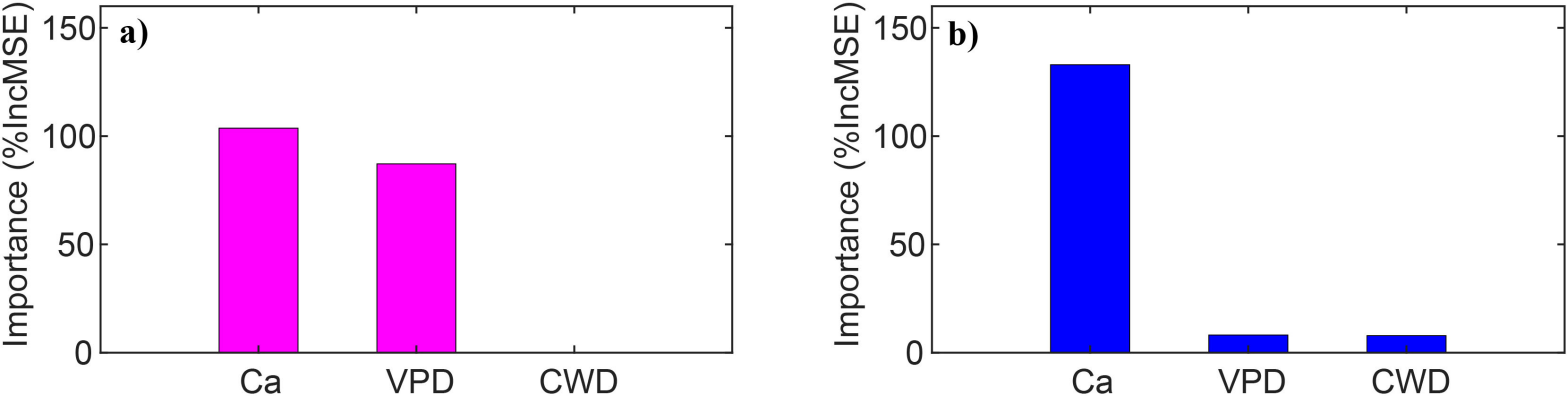
Relative importance of atmospheric CO_2_ (Ca), vapor pressure deficit (VPD), climatic water deficit (CWD) to intrinsic water use efficiency (iWUE) via the random forest analysis at dry **(a)** and wet **(b)** sites. %IncMSE refers to increase in mean squared error in the random forest analysis.

iWUE increased significantly (p<0.01) at both dry and wet sites over the past three decades, with dry sites exhibiting a slightly higher rate of increase (**Figure 3**). Linear mixed-effects models explained 84% and 82% of iWUE variance at dry and wet sites, respectively, revealing strong positive relationships with atmospheric CO_2_ and VPD (**Table 3**). Across all sites, iWUE consistently increased with rising atmospheric CO_2_ throughout the observed VPD range (0.1–1.2 kPa) (**Figure 4**). Random forest analysis showed that the importance of atmospheric CO_2_ was the 103.65% increase in mean squared error at dry sites, and 132.93 at wet sites, which was quite higher than those of VPD (87.18% for dry sites and 8.17% for wet sites) and climatic water deficit (0.11% for dry sites and 7.89% for wet sites) (**Figure 5**). Notably, climatic water deficit showed a significant effect on iWUE at the wet site during 1980–2015 (**Table 3**).

**Table 3:** Parameter estimates for the linear mixed-effects model examining the drivers of intrinsic water use efficiency (iWUE) and basal area increment (BAI) for the period 1980–2015.

Group		iWUE				BAI			
		Estimate	SE	t-value	p-value	Estimate	SE	t-value	p-value
Dry sites	Intercept	0	0.32	0	1.00	0	0.57	0	1
	Ca	0.70	0.05	13.72	<0.01	0.27	0.07	3.85	<0.01
	VPD	0.21	0.08	2.61	<0.01	−0.33	0.21	−1.57	0.12
	CWD	−0.11	0.07	−1.77	0.08	0.11	0.10	1.06	0.29
Wet sites	Intercept	−0.003	0.22	−0.02	0.99	−0.003	0.38	−0.01	0.99
	Ca	0.81	0.05	16.04	<0.01	0.29	0.07	4.18	<0.01
	VPD	0.21	0.10	2.00	<0.05	−0.49	0.14	−3.42	<0.01
	CWD	−0.19	0.07	−2.80	<0.01	−0.17	0.09	−1.95	0.05

Ca is atmospheric CO_2_, VPD is vapor pressure deficit, and CWD is climatic water deficit.

### 3.3 Temporal trends in BAI and environmental responses

**Figure 6:**
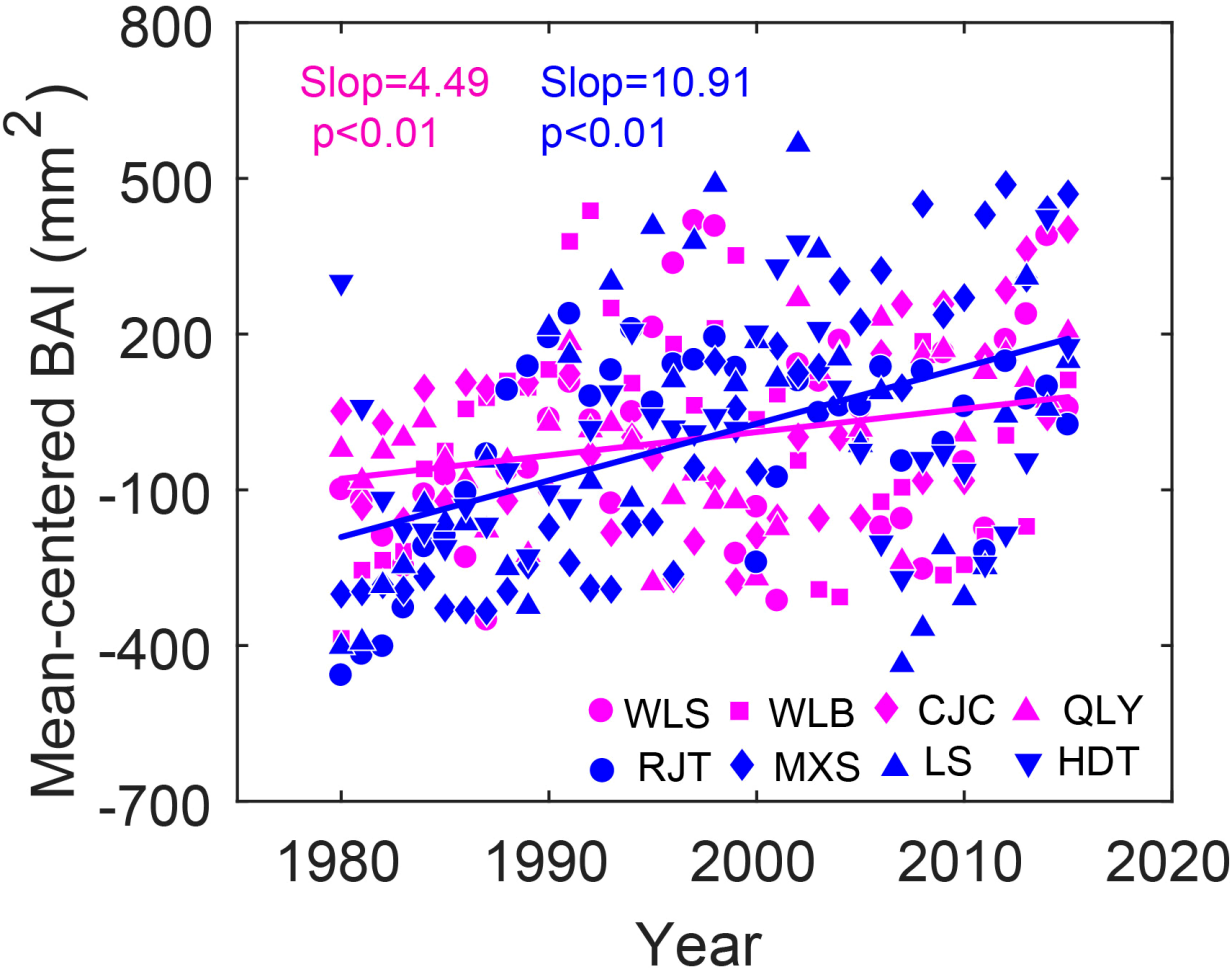
Temporal variations in the mean-centered basal area increment (BAI) of Chinese pine in eight sites over northern China for the period of 1980–2015. Red colors represent dry sites, whereas blue colors represent wet sites. Different symbols represent different sites. The lines are the linear fits.

BAI of both dry and wet sites increased with years in the periods of 1980-2015 (p <0.01), with a little lower increase rate in dry sites (**Figure 6**). Linear mixed-effects models showed that atmospheric CO_2_ was positively correlated with the variation of BAI at all sites, whereas VPD and climatic water deficit were significantly related to BAI at wet sites (**Table 3**).

### 3.4 Temporal trends in leaf water δ^18^O enrichment above the source water

**Figure 7:**
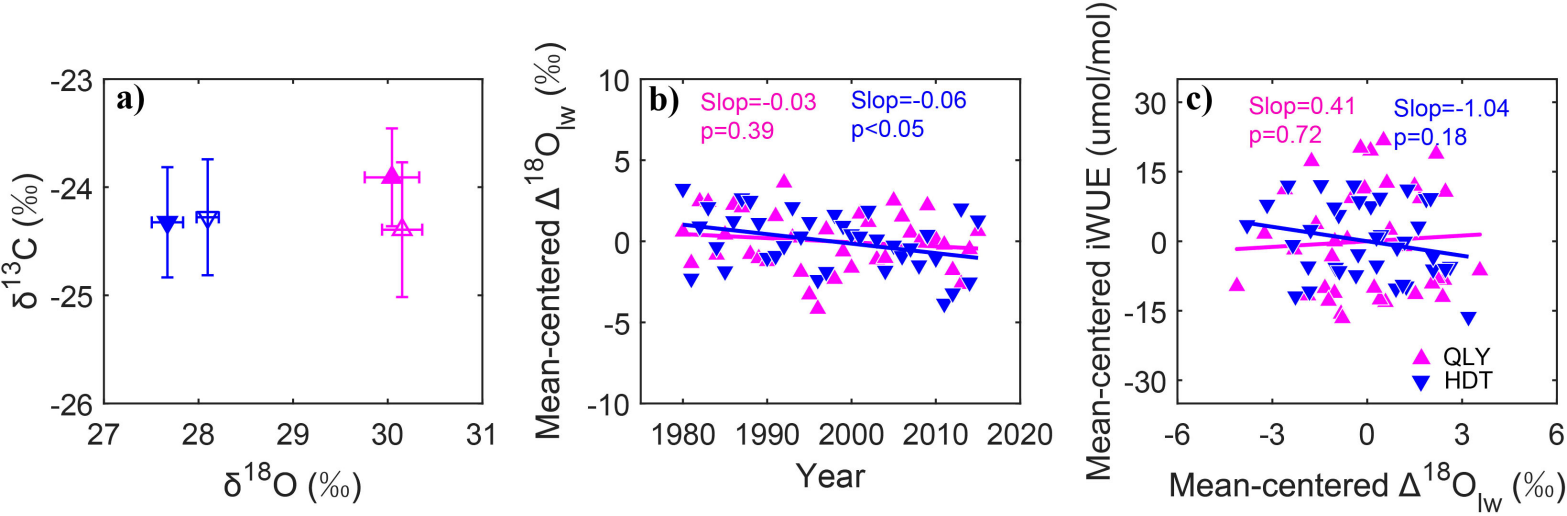
Shifts of stable isotopic signatures (δ^13^C and δ^18^O, **a**) between the years of 1980–1997 (open symbols) and 1998–2015 (filled symbols), and temporal variations of leaf water δ^18^O enrichment above source water (Δ18Olw, **b**) of Chinese pine over northern China for the period of 1980-2015, and relationship between intrinsic water use efficiency (iWUE) and leaf water δ^18^O enrichment above source water (Δ^18^O_lw_, **c**). Red color represents dry sites, while blue color represents wet sites. Error bars represent the standard deviations of observations. The lines are the linear fits.

Variation of stomatal conductance was further analyzed using long-term stable δ^18^O chronologies from QLY (dry site) and HDT (wet site), given the significant iWUE increases at both dry and wet sites. Dual-isotope analyses revealed variations in tree ring δ^13^C and δ^18^O values between 1980–1997 and 1998–2015 (**Figure 7a**). Tree ring δ^13^C composition increased at the dry site, whereas it remained constant at the wet site. Tree ring δ^18^O composition decreased at both dry and wet sites. The isotopic shift indicated enhanced net photosynthesis accompanied by a constant or increased stomatal conductance based on the dual-isotope conceptual model (Scheidegger et al., 2000). Furthermore, leaf water δ^18^O enrichment above the source water showed no significant temporal trend at the dry site (p>0.05), suggesting minimal stomatal conductance changes. Conversely, at the wet site, leaf water δ^18^O enrichment above the source water showed a decreasing trend, corresponding to increased stomatal conductance (**Figure 7b**). Accordingly, iWUE and leaf water δ^18^O enrichment above the source water exhibited positive correlation at the dry site, and negative correlation at the wet site (**Figure 7c**).

## 4 Discussion

### 4.1 Environmental factors affecting iWUE

Consistent with our first hypothesis, rising atmospheric CO_2_ exerted a dominant positive influence on iWUE in Chinese pine, overriding drought effects at both dry and wet sites. Elevated CO_2_ enhances BAI, which is related to photosynthesis rate (**Table 3**; **Figure 6**). Given that iWUE is the ratio of assimilation rate to stomatal conductance, our isotopic evidence indicates an increased assimilation rate with stable (dry sites) or increased (wet sites) stomatal conductance for Chinese pine (**Figure 7**). This demonstrates that stimulated photosynthesis is the primary driver of increased iWUE across the precipitation gradient—a pattern corroborated by tree ring δ^13^C and δ^18^O in multiple species (Guerrieri et al., 2019; Mathias and Thomas, 2021). This finding rejected our third hypothesis. Similar findings were also reported in other studies using different methods that show global increases in assimilation rate as a result of rising atmospheric CO_2_ (Chen et al., 2022; Keenan et al., 2023). However, this result challenges the assumption that reductions in stomatal conductance led to increased iWUE in other tree species, like Qinghai spruce at the northeastern Qinghai–Tibetan plateau (Wang et al., 2021b) and Scots pine in Europe (Martinez-Sancho et al., 2018). In these cases, drought emerges as the primary influencing factor on iWUE and the enhancement of iWUE is likely attributed to a reduction in stomatal conductance rather than an increase in assimilation rate.

VPD can modulate iWUE even in the absence of significant soil moisture variation at both dry and wet sites, demonstrating its primary role in iWUE regulation compared with soil moisture. This result aligns with findings across diverse ecosystems, including deciduous broadleaf forests and evergreen needleleaf forests (Novick et al., 2016; Yi et al., 2019; Zhang et al., 2019). Globally, VPD has increased exponentially in recent decades due to rising temperature. Under conditions of high VPD, plants typically reduce stomatal conductance to prevent excessive water loss and critical xylem tension. This stomatal closure constrains photosynthesis (Yuan et al., 2025). Transpiration initially increases with high VPD until it reaches a threshold, beyond which it plateaus or declines, intensifying tree water stress (Novick et al., 2024). Therefore, the chronic, global, temperature-driven increase in VPD will increasingly govern vegetation productivity, hydrologic cycles, and climate feedbacks in the coming decades (Bauman et al., 2022; Trotsiuk et al., 2021).

At wet sites, decreased soil moisture had a significant impact on iWUE, reflecting the variation of iWUE co-regulated by both VPD and soil moisture. This finding was inconsistent with our second hypothesis. The superior soil water availability at wet sites facilitates higher stomatal conductance, which in turn enhances the assimilation rate (Wang et al., 2021a). Moreover, the capacity for seasonal shifts in water uptake at humid sites compensated for the detrimental effects of seasonal drought on tree growth (Li et al., 2022). Consequently, soil water availability emerges as a critical factor driving iWUE dynamics specifically at wet sites.

### 4.2 Stomatal regulation

Stronger stomatal regulation is observed at the dry site (QLY) than at the wet site (HDT), although our results did not support our third hypothesis. Temporal trends in stomatal conductance derived from leaf water δ^18^O enrichment above the source water chronologies reveal that stomatal conductance remained constant at the dry site, compared with increased stomatal conductance at the wet site (**Figure 7**). Tighter stomatal control over iWUE in Chinese pine in moisture-limited conditions aligns with previous reports (Li et al., 2023) and reflects a critical hydraulic strategy: greater stomatal sensitivity to reduced soil-to-leaf hydraulic conductance hydraulically buffers the vascular system against dry soil, delaying hydraulic failure and dehydration (Li et al., 2019).

The rising stomatal conductance observed in the trees at the wet site can potentially amplify transpiration water loss, thereby significantly impacting hydrologic cycles. The increased transpiration by Chinese pine plantations can contribute to elevated humidity and an enhanced recycling of precipitation (Tian et al., 2022). However, the extent to which hydrologic cycles have already been altered due to increased transpiration remains unclear, as these changes have occurred simultaneously with other factors such as forestation. These interconnected phenomena warrant extensive research attention to better understand their implications (van der Sleen et al., 2014; Zhang et al., 2022).

The physiological mechanisms governing stomatal responses to environmental change remain complex and incompletely resolved. First, while rising atmospheric CO_2_ is expected to close stomata to minimize water loss without reducing carbon uptake (Liang et al., 2023), it may simultaneously reduce stomatal sensitivity to drought (Lauriks et al., 2022). Second, it is difficult to distinguish the role of VPD and soil moisture on stomatal response because both influence stomatal conductance through leaf turgor pressure (Buckley, 2017). Further studies like control experiments are needed to isolate their individual impacts. Third, methodological debates persist regarding stomatal conductance estimates from tree ring δ^18^O (Lin et al., 2022; Roden and Siegwolf, 2012). In addition, our study included only two sites with tree ring δ^18^O analyses, which limited our ability to infer differences in stomatal regulation across the broader site network. Despite these uncertainties, our core findings remain robust that rising atmospheric CO_2_ enhances the assimilation rate and increases iWUE in Chinese pine across precipitation gradients.

## 5 Conclusion

In summary, conducting a comparative analysis of Chinese pine under two distinct climate conditions has significant implications for assessing tree iWUE responses to environmental changes. Our examination of the relative impacts of various environmental drivers revealed the significance of rising atmospheric CO_2_ and elevated VPD in regulating tree iWUE, especially the substantially positive effect of atmospheric CO_2_ on carbon fixation, which amounted for increased iWUE. Our findings clarify the environmental mechanisms regulating the long-term iWUE variation and will aid in defining this tree species’ performance under climate change in northern China.

## Data Availability

The original contributions presented in the study are included in the article/supplementary material. Further inquiries can be directed to the corresponding authors.
